# Hierarchy of cellular decisions in collective behavior: Implications for wound healing

**DOI:** 10.1038/srep20139

**Published:** 2016-02-02

**Authors:** Lisa E. Wickert, Shaun Pomerenke, Isaiah Mitchell, Kristyn S. Masters, Pamela K. Kreeger

**Affiliations:** 1 Department of Biomedical Engineering, University of Wisconsin-Madison, 1550 Engineering Dr, Madison, WI, 53706 United States of America

## Abstract

Collective processes such as wound re-epithelialization result from the integration of individual cellular decisions. To determine which individual cell behaviors represent the most promising targets to engineer re-epithelialization, we examined collective and individual responses of HaCaT keratinocytes seeded upon polyacrylamide gels of three stiffnesses (1, 30, and 100 kPa) and treated with a range of epidermal growth factor (EGF) doses. Wound closure was found to increase with substrate stiffness, but was responsive to EGF treatment only above a stiffness threshold. Individual cell behaviors were used to create a partial least squares regression model to predict the hierarchy of factors driving wound closure. Unexpectedly, cell area and persistence were found to have the strongest correlation to the observed differences in wound closure. Meanwhile, the model predicted a relatively weak correlation between wound closure with proliferation, and the unexpectedly minor input from proliferation was successfully tested with inhibition by aphidicolin. Combined, these results suggest that the poor clinical results for growth factor-based therapies for chronic wounds may result from a disconnect between the individual cellular behaviors targeted in these approaches and the resulting collective response. Additionally, the stiffness-dependency of EGF sensitivity suggests that therapies matched to microenvironmental characteristics will be more efficacious.

Biological processes such as wound healing, morphogenesis, and metastasis result from the action of individual cells that together produce a collective behavior. This sets up an inherently challenging problem when designing therapies - while directing the collective behavior is the ultimate goal, this is accomplished by correctly regulating the behaviors of individual cells. Therefore, to control collective behaviors, it is necessary to first determine which individual cellular behaviors contribute to the collective response, and then define their relative contributions such that the therapy is designed to target the most significant behaviors in the most efficacious way. However, empirically deciphering the relative contribution of different individual cell behaviors to the collective response is a challenging task and remains unknown for most biological processes.

One application for such knowledge is in the design of therapies to regulate dermal wound healing, where keratinocytes act in a collective manner to re-epithelialize the wound^1^,^2^. During the course of re-epithelialization, keratinocytes migrate as a cell sheet over the granulation tissue produced by fibroblasts and differentiate to reform the barrier of the skin^3^,^4^. Failure of this re-epithelialization process results in the development of a chronic wound, which affects 6.5 million individuals in the U.S. and costs $25 billion annually^5^. Wound dressings that deliver biomolecules to increase keratinocyte proliferation and migration speed have been explored for over a decade, based on the assumption that these two behaviors are the dominant contributors to collective displacement of the keratinocyte sheet. However, these biomolecule-delivering wound dressings have not been successful in the clinic^6^,^7^, suggesting the need to evaluate which cellular behaviors should be targeted for improved healing.

In the current study, we characterized collective wound closure and the underlying individual cell behaviors of proliferation, cell migration, and spreading for keratinocytes stimulated with epidermal growth factor (EGF), a potent regulator of keratinocyte proliferation, migration, and differentiation^8^, across a range of mechanics found in the wound microenvironment^9^,^10^,^11^. The resulting data set was analyzed by partial least squares regression (PLSR)^12^ to characterize the differential contributions of individual cell behaviors to collective migration. Our findings suggest that current biomolecule delivery strategies do not target the cell behaviors needed to optimally increase re-epithelialization.

## Results

### Combined effects of substrate stiffness and EGF stimulation on collective migration

Re-epithelialization is not the result of migration of isolated cells, but rather the product of collective displacement of keratinocytes from the wound edge. EGF, which is released primarily by platelets, macrophages, and fibroblasts following injury, has long been studied in the context of wound re-epithelialization and regulates keratinocyte proliferation, migration, and differentiation^8^,^13^. In addition, as with most complex physiological processes, re-epithelialization is influenced by other elements of the cellular microenvironment. Previous research has shown that the mechanics of this environment affect keratinocyte behavior, with stiffer substrates promoting collective migration and cell proliferation^14^. The elastic modulus of dermal wounds is dynamic, ranging from 1-20 kPa immediately after injury to >100 kPa by one week post-wounding^9^,^10^,^11^. Therefore, we utilized combinations of these two external cues to provide a range of stimuli and analyze how individual keratinocyte behaviors integrate to regulate wound closure.

Keratinocytes (HaCaTs) were plated on polyacrylamide (PAA) gels of three stiffnesses (1, 30, and 100 kPa, Figure S1A) conjugated with equal concentrations of collagen (Figure S1B), stimulated with four doses of EGF (0, 0.1, 1, and 10 ng/mL), and imaged every day for 4 days to calculate percent wound closure relative to day 0 (Figure S2). Western blots for phosphorylated EGFR confirmed that EGF treatment stimulated EGFR phosphorylation in cells cultured on each of the three stiffnesses (Figure S3A) and that the magnitude of the response was dependent upon EGF dose (Figure S3B).

**Figure 1:**
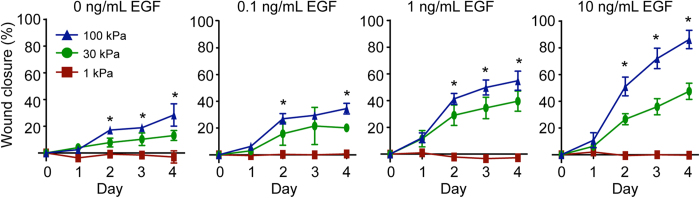
Collective migration of keratinocytes. Wound closure relative to day 0 for a fence migration assay performed on 1, 30, and 100 kPa PAA gels treated with increasing doses of EGF. Data presented as average ± SD, n = 3. *indicates p < 0.05 for 100 kPa vs. 30 kPa on the same day and EGF dose.

With respect to wound closure, a stiffness-dependent increase in closure was observed in the absence of EGF, with cells on 100 kPa displaying the highest wound closure compared to cells on 1 and 30 kPa substrates (Fig. 1). This difference was apparent as early as day 2 and continued through the end of the experiment on day 4. The cellular responsiveness to EGF treatment was also dependent upon substrate stiffness. Notably, keratinocytes on the softest condition (1 kPa) were unresponsive to EGF stimulation, with the highest dose of EGF unable to increase wound closure relative to the untreated 1 kPa control. In contrast, wound closure on 30 and 100 kPa substrates each demonstrated a dependence upon EGF dose and displayed significantly higher closure rates compared to 1 kPa substrates at all EGF doses (p < 0.05). Treatment with 10 ng/mL EGF further augmented the difference in wound closure between 30 and 100 kPa substrates. Due to the limited collective migration capacity of keratinocytes on 1 kPa substrates, the remainder of this study focused on the individual behaviors driving wound closure on 30 and 100 kPa substrates.

### Single cell speed and persistence during collective migration

**Figure 2:**
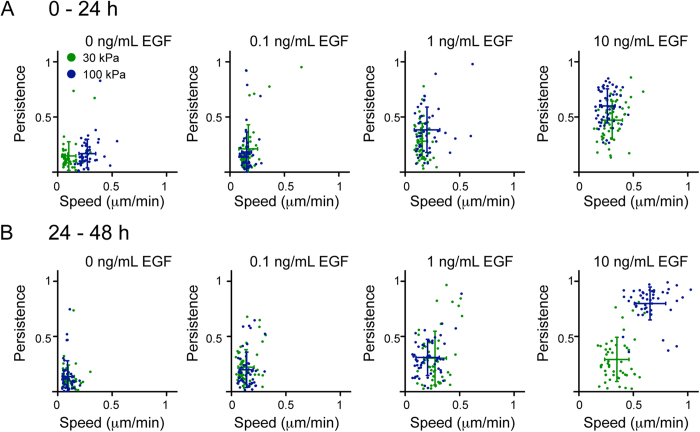
Speed and persistence of individual keratinocytes during collective migration. Cell speed vs. persistence for individual cells tracked for (**A**) 0–24 h and (**B**) 24–48 h after fences were lifted on 30 and 100 kPa PAA gels treated with increasing doses of EGF. Data presented as a point for each individual cell, error bars represent population average (intersection) and SD, n = 50 for each condition.

To determine if the different individual cellular processes involved in collective migration are also impacted by differences in stiffness and EGF, we first quantified speed and persistence for single cells located at the wound edge. In collective migration, these leading cells migrate into the open space and ‘pull’ the cells from the bulk to move with them^15^. Our initial observations suggested that individual cell migration differed over time when examined in the context of collective migration. From 0–24 hours, individual cell speed was slightly higher on 100 kPa gels versus 30 kPa gels in the absence of EGF, while persistence was unaffected (Fig. 2A). The addition of EGF resulted in a loss in stiffness-dependence with respect to speed, and a significant increase in persistence that was further increased on the stiffer substrate (p < 0.05; Table S1). In contrast, from 24–48 hours there was no difference with respect to stiffness for speed or persistence in the absence of EGF (Fig. 2B). Upon treatment with EGF, cells on both 30 and 100 kPa substrates were observed to migrate faster and with increased persistence. A stiffness-dependent effect was observed only when cells were treated with 10 ng/mL EGF; similar to the wound closure results for this condition (Fig. 1), cells migrated significantly faster and with significantly higher persistence on 100 kPa gels than on 30 kPa gels (p < 0.05).

An examination of keratinocytes plated as isolated cells yielded starkly different findings with respect to migration behaviors (Figure S4). Specifically, in contrast to single cells within a collective sheet, isolated cells did not exhibit increased persistence with either increased substrate stiffness or EGF dose. Additionally, isolated cells on the 100 kPa substrate displayed significantly reduced speeds compared to 30 kPa substrate when treated with 10 ng/mL EGF (p < 0.05). These results are consistent with existing literature on isolated cell behavior^16^,^17^, but the opposite of the behavior observed for cells in the collective population.

### Proliferation in the bulk and along the wound edge during collective migration

**Figure 3:**
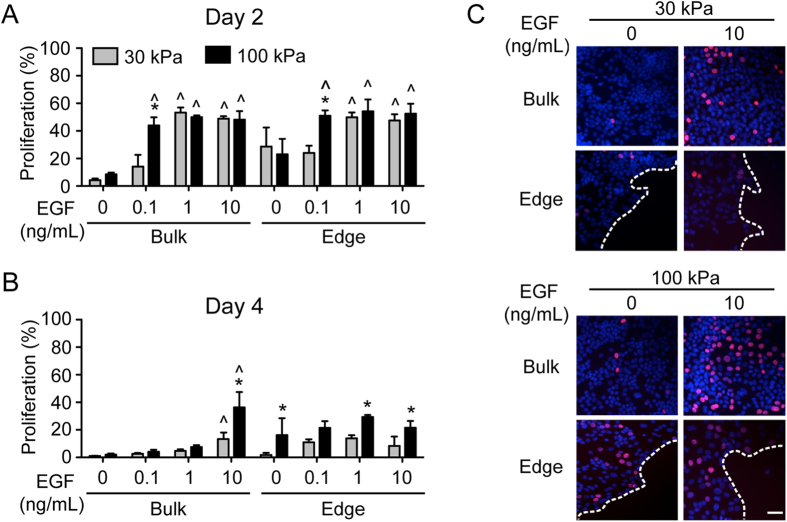
Bulk and edge proliferation of keratinocytes. Proliferation indices quantified at the wound bulk and edge at (**A**) day 2 and (**B**) day 4 on 30 and 100 kPa PAA gels with increasing doses of EGF. Data presented as average ± SD, n  = 3, * indicates p < 0.05 compared to 30 kPa at the same dose, ^ indicates p < 0.05 compared to 0 ng/mL EGF at the same stiffness. (**C**) Representative images for day 4 proliferation. Red indicates EdU and blue is DAPI nuclei stain; dashed line indicates wound edge. Scale bar = 50 μm.

Keratinocyte proliferation is observed during wound healing *in vivo,* and it is well established that keratinocytes proliferate *in vitro* in response to EGF treatment or substrate stiffness^14^,^18^. Therefore, we next examined proliferation during our collective migration assay, looking at the proliferation of cells in the bulk and along the leading edge. Similar to the observed time-dependent effects for speed and persistence, patterns of keratinocyte proliferation were different for day 2 and day 4 of the experiment (Fig. 3). On day 2 (Fig. 3A), cell proliferation in the absence of EGF was similar between the 30 and 100 kPa conditions, but was significantly higher at the wound edge. Treatment with EGF stimulated proliferation of both bulk and edge cells, and cellular sensitivity to EGF dose increased with substrate stiffness. Specifically, a dose of 0.1 ng/mL EGF was sufficient to increase both bulk and edge proliferation on 100 kPa substrates, while 1 ng/mL EGF was needed to increase either bulk or edge proliferation on the 30 kPa condition.

Proliferation at day 4 (Fig. 3B) was generally lower compared to day 2 and higher along the wound edge compared to the bulk for the same conditions. In the bulk, only cells that were treated with the highest dose were sensitive to EGF; similar to day 2, this sensitivity was increased on the stiffer substrate. Cells on the edge at day 4 did not appear to be sensitive to EGF but did demonstrate sensitivity to stiffness, exhibiting increased proliferation on 100 kPa substrates relative to the 30 kPa condition.

### Cell area in the bulk and at the wound edge during collective migration

**Figure 4:**
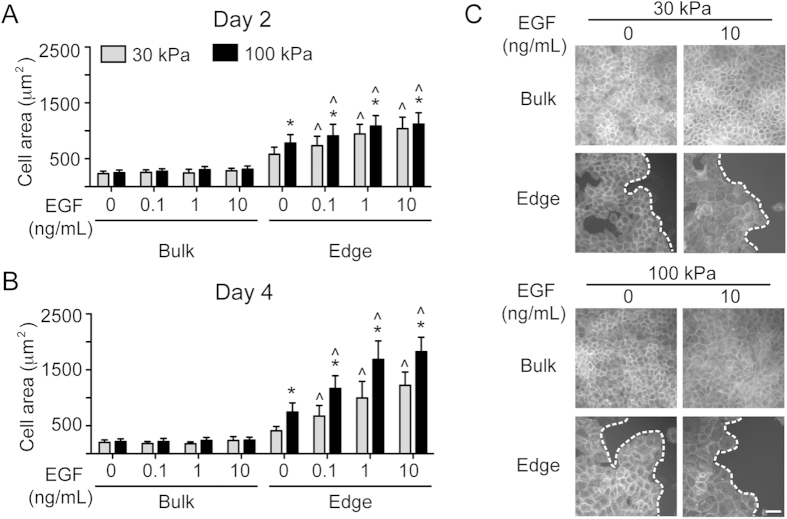
Bulk and edge area of keratinocytes. Cell area quantified in the wound bulk and at the edge at (**A**) day 2 and (**B**) day 4 on 30 and 100 kPa PAA gels with increasing doses of EGF. Data presented as average ± SD, n = 30, *indicates p < 0.05 compared to 30 kPa at the same EGF dose, ^indicates p < 0.05 compared to 0 ng/mL EGF at the same stiffness. (**C**) Representative phalloidin images at day 4 of collective migration; dashed line indicates wound edge. Scale bar = 50 μm.

In addition to migration and cell proliferation, changes in cell size may contribute to total wound closure as cells spread into the available space. Therefore, we analyzed cell area for both bulk and leading edge keratinocytes during collective migration. Bulk cell area was unresponsive to EGF dose and substrate stiffness at both days 2 and 4 (Fig. 4A,B). In contrast, cell area along the leading edge increased on 100 kPa substrates compared to 30 kPa substrates at 0 ng/mL EGF at both day 2 and day 4. This trend was maintained at each EGF dose, where cells at the wound edge on 100 kPa gels were significantly larger compared to cells on 30 kPa gels. Additionally, cell area along the leading edge demonstrated an EGF dose-dependency on both 30 and 100 kPa gels. This hypertrophy was further increased at day 4 relative to day 2 for cells on the 100 kPa condition. Despite the large increase in cell area, cell morphology remained normal across all conditions at day 4 (Fig. 4C).

### PLSR model analysis of relationship between individual cell behaviors and wound closure

**Figure 5:**
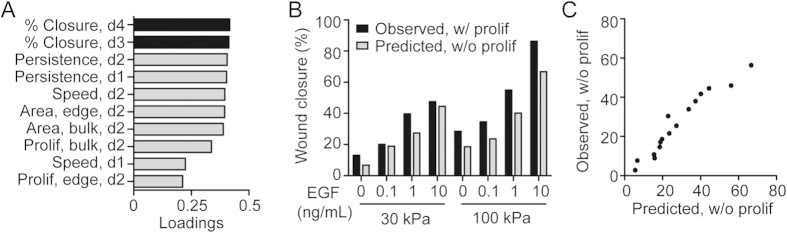
PLSR model for collective migration of keratinocytes. (**A**) Variable loadings for the independent (gray) and dependent (black) variables. (**B**) Wound closure (%) at day 4 observed in model training set with proliferation (black) vs. model predicted effect of complete inhibition of proliferation (gray). (**C**) Wound closure (%) at day 3 and 4 observed when proliferation was inhibited with 4 μg/mL aphidicolin vs. model prediction.

While individual cell behaviors appeared to potentially explain the differences in collective migration in response to substrate stiffness and EGF concentration, the observed changes in individual cell migration, proliferation, and area did not correlate precisely, suggesting that multiple cell behaviors might interact to regulate the collective response. To analyze how these individual cell decisions interact to regulate the collective response, we utilized PLSR^19^, a multivariate regression technique that has previously been used to analyze a variety of relationships^20^,^21^,^22^. To create the model, cell behaviors on days 0–2 were compiled to predict keratinocyte behaviors at days 3 and 4. A single component model (Figure S5A) showed a strong correlation (R^2^Y = 0.94) between individual and collective behaviors, and was predictive by cross-validation (Q^2^Y = 0.86). To understand how the model interprets the relationship between individual and collective behaviors we examined the loadings, which describe the direction and magnitude that each variable projects along the principal components (Fig. 5A). The values of the loadings are a result of the PLSR algorithm, in which independent (**X**) variables with similar variation to dependent (**Y**) variables will have a similar loading. There were a number of variables that strongly correlated with wound closure at both days 3 and 4, including cell area, single cell persistence, and day 2 cell speed. Indeed, edge cell area closely followed the general collective migration trends, with increases in response to both EGF and stiffness (Fig. 4). The loading for persistence reflects that persistence was the only individual cell behavior that demonstrated an increase for the 100 kPa and 10 ng/mL EGF condition with respect to both stiffness and EGF dose (Fig. 2), and a similar interaction was observed in wound closure for this combination (Fig. 1). Interestingly, initial speed (0–24 h) and cell proliferation, both in the bulk and at the wound edge, were not strongly correlated with wound closure in the model.

To examine the hypothesis that proliferation was not essential for the observed differences in wound closure, the PLSR model was used to predict the quantitative impact of complete inhibition of proliferation on collective wound closure at days 3 (Figure S5B) and 4 (Fig. 5B). When proliferation was inhibited in the model, wound closure was predicted to decrease by no more than 20% compared to wound closure when proliferation was present; additionally, the relationship between cell migration behaviors and increasing EGF dose and/or substrate stiffness was predicted to be maintained in the absence of proliferation. To test this model prediction experimentally, keratinocyte proliferation was inhibited via application of aphidicolin during collective migration. Aphidicolin treatment successfully inhibited proliferation, as evidenced by Click-iT EdU staining of the 10 ng/mL EGF condition at multiple time points (Figure S6A). Compared to vehicle-treated cells, aphidicolin-treated cells displayed lower total wound closure by day 4 on both 30 and 100 kPa substrates (Figure S6B,C), and the magnitude of this decrease in closure was predicted by the model with good accuracy (Fig. 5C, R^2^ = 0.93). Moreover, as predicted by the model, the relative trends of increasing closure with increasing stiffness and EGF dose were preserved. Taken together, these observations confirm that the model correctly predicted the contribution of proliferation to overall wound closure.

## Discussion

The integration of individual cellular decisions to result in collective migration of keratinocytes is critical for wound re-epithelialization. Although keratinocytes exist only as a collective *in vivo*, most studies of individual keratinocyte speed and persistence have examined isolated cells. The findings reported herein reveal new information about these collective behaviors and illustrate that isolated and collective cells can respond very differently to the same stimuli. In order to determine which of these individual cellular behaviors may represent potential future targets for therapeutic intervention, we characterized how combinations of EGF dose and substrate stiffness impacted keratinocyte collective migration and utilized this information to predict how changes in individual cellular decisions impact this collective response. This combined experimental and computational research strategy yielded results that have potential implications for understanding the failures of existing wound treatments as well as designing new approaches that provide appropriate environments (*e.g.*, wound dressing mechanics) and stimuli (*e.g.*, soluble factors) to impact wound healing.

Re-epithelialization results from the integration of individual cellular processes, including proliferation, migration, and spreading. Previous work has shown that the proliferation of isolated keratinocytes can be promoted by the delivery of EGF or by changing substrate stiffness^14^,^23^. Using a model of collective migration, our study revealed more nuanced information about keratinocyte proliferation in response to these stimuli. Namely, cell proliferation in the bulk and edge were differentially affected by substrate stiffness and EGF dose (Fig. 3), with bulk proliferation more responsive to EGF dose and edge proliferation more responsive to substrate mechanics on day 4 of wound closure. Despite these effects, the PLSR model indicated that cell proliferation was not a driving factor in the differences in wound closure in response to different stiffnesses and EGF doses (Fig. 5), suggesting that the failure of growth factor delivery systems for wound healing may be because they are not targeting the cellular behaviors that are the most important for sustained re-epithelialization. This is perhaps not surprising as there is evidence that keratinocytes at the wound edge of chronic wounds are hyperproliferative, but non-migratory^24^, indicating that proliferation alone does not support re-epithelialization *in vivo*. To test the prediction that proliferation is not essential *in vitro*, we examined the extent of wound closure in response to aphidicolin treatment and found that inhibition of proliferation did not ablate wound closure (Fig. 5C). While the absolute level of wound closure was slightly decreased across all conditions, the relative effects of different stiffnesses and EGF doses were preserved, supporting the conclusion that, while mitogenic-focused approaches can partially support wound healing, targeting other cellular behaviors may provide a more robust response.

Instead, PLSR analysis indicated that the individual cell behaviors that were the most correlated with collective wound closure were cell migration persistence and cell area. Increased substrate stiffness and EGFR overexpression have previously been shown to promote increased migrational persistence of other epithelial cell types during collective migration^25^,^26^. It is perhaps not surprising that persistence would play an important role in determining wound closure, as migration of individual keratinocytes without persistent movement into the wound would not accomplish complete re-epithelialization. More intriguing is the suggestion that cell area strongly influenced wound closure. Several previous studies have demonstrated that the spreading of isolated cells increased with substrate stiffness^27^,^28^. However, our examination of keratinocytes cultured in a collective sheet revealed that only edge cells were sensitive to substrate mechanics, while bulk cell area was not affected by substrate stiffness. This observation is consistent with keratinocyte hypertrophy in native wound healing being limited to only the leading edge, and coupled with maintenance of a high-density sheet of smaller cells in the bulk population^29^. The substantial (~8-fold) increase in edge cell area observed in some conditions was accompanied by the maintenance of normal morphology, and hypertrophied cells continued to be migratory and moderately proliferative. This behavior is consistent with *in vivo* keratinocyte function, where these cells may undergo extensive hypertrophy during normal physiological processes^30^. While the PLSR model was built using unit-variance scaled data to enable comparisons of cellular behaviors that are measured on different scales, calculations using the absolute magnitudes of the cell and wound areas suggest that the extent of hypertrophy observed in these experiments could substantially contribute to wound closure. For example, the difference in average edge cell area on 30 vs. 100 kPa substrates equated to the hypertrophy of only 45,000 cells (6% of cells originally plated) to account for the difference in wound closure between these conditions in the absence of any proliferation or migration. Overall, these findings highlight the underappreciated, yet potentially significant, role of keratinocyte hypertrophy in achieving wound re-epithelialization. However, while robust differences in both collective and individual behaviors were found across variations in both stiffness and EGF dose in this work, *in vivo* studies will be needed to determine if these differences translate into biologically significant outcomes. Likewise, it will be important to validate the hierarchy of individual cell behaviors described in the current work; if the *in vivo* effects are consistent with the *in vitro* model of collective migration, this would suggest that biomolecules and biomaterials that promote cell spreading should be utilized for improved wound healing.

Our results suggest another possible explanation for the failures with trials of growth factor delivery for treatment of chronic wounds. While recent studies have suggested a link between growth factor sensitivity and substrate mechanics^31^,^32^,^33^, this phenomenon has not previously been examined in the context of dermal wound healing, and the impact on collective cell environments remains particularly understudied. Here, we observed that EGF enhanced wound closure on 30 and 100 kPa gels (Fig. 1), but cells on 1 kPa gels were insensitive to even the highest dose of EGF and were unable to close the wound. Early stage wounds have a stiffness of 1–20 kPa immediately after injury^9^,^10^,^11^; therefore, the clinical failure of EGF-based wound dressings may be a case of a mismatch between the chosen stimuli and keratinocyte sensitivity due to other stimuli from the microenvironment. Our examination of EGFR activation found that EGFR was phosphorylated across the three different substrates (Figure S3A), demonstrating that receptor-level activation is necessary, but not sufficient, for changes in cellular- and population-level phenotypes. Importantly, wounds increase in stiffness over time in animal models^9^,^34^, suggesting that EGF therapy could be more effective if applied at later stages of wound healing. These results highlight the importance of developing therapies that are context-specific; in a dynamic process such as wound healing this may require the development of distinct therapies for different stages.

Given the complexity of biological processes such as wound healing and the large design space for biomaterials-based methods to treat these conditions, computational analysis is emerging as an important tool to determine optimal approaches. PLSR has previously been utilized to determine the optimal chemical and physical characteristics of biomaterials in order to induce cellular behaviors^20^,^35^ and to analyze how signaling events mediate the effects of different biomaterials design approaches^36^. Here, we extended the use of PLSR to a new application in order to determine how changes to individual cell behaviors integrate to control the impact on collective behaviors. This approach – to deconstruct the collective response, decode the contributions of individual cellular behaviors, and then identify methods to target them – could be broadly adapted to aid in the design of therapeutic strategies. Our results suggest that future biomaterials-based approaches to wound healing should focus on enhancing keratinocyte persistence and spreading. Additionally, while clinical success of growth factor-releasing wound dressings has been elusive and hindered by obstacles such as high cost and oncogenic potential due to the delivery of large amounts of growth factor^7^, we postulate that sustained growth factor delivery to wounds may not be warranted. Specifically, in the case of EGF, delivery may be beneficial only during the later stages of wound healing.

## Materials and Methods

### Reagents

Reagents were purchased from Sigma-Aldrich (St. Louis, MO) unless otherwise noted.

### Fabrication and characterization of polyacrylamide substrates

Polyacrylamide (PAA) gel substrates were fabricated via photopolymerization as described previously^37^,^38^. Briefly, a final solution of 5%, 15%, or 25% (w/v) acrylamide and 0.2% (w/v) bisacrylamide was prepared in 0.5% (w/v) Ciba® Irgacure®-2959 (BASF, Germany) to obtain gel substrates of 1, 30, and 100 kPa, respectively. Dynamic mechanical analysis (MTS Insight 5) was performed on PAA disks 2 mm in thickness and 8 mm in diameter, and the elastic modulus was calculated from the stress-strain curve at 1 Hz at room temperature.

For all other studies, PAA gels were polymerized on 20 mm silanized glass coverslips (Marienfield-Superior, Germany) as described^37^. Briefly, 20 μL of prepolymer solution was pipetted onto a parafilm stage, and the silanized coverslip was carefully applied onto the prepolymer drop. The sandwich system was exposed to UV at 365 nm for 5 min using an UVP Black-Ray lamp (Model XX-15L, 115 v, 60 Hz, Upland, CA), and gels were allowed to swell in PBS overnight. To facilitate cell attachment, gels were functionalized with 1 mg/mL sulfosuccinimidyl 6-(4′-azido-2′-nitrophenylamino) hexanoate (sulfo-SANPAH, ThermoFisher Scientific, Waltham, MA) in 50 mM HEPES pH 8.0 buffer and exposed to UV at 365 nm for 10 min. Sulfo-SANPAH-conjugated gel surfaces were then rinsed with PBS three times and adhered to 12-well tissue culture plates (Corning, Tewksbury, MA) by applying the 100 kPa prepolymer solution to the underside of the coverslip and exposing to UV at 365 nm for 5 min. Gels were then incubated with 0.2 mg/mL PureCol® bovine type I (Advanced Biomatrix, San Diego, CA) diluted in 0.1% (v/v) acetic acid overnight at 4°C, rinsed in PBS three times, and UV sterilized at 254 nm for 1 h before use.

### Cell culture

Immortalized human keratinocytes (HaCaTs) were courtesy of N. Fusenig (DKFZ, Heidelberg, Germany). HaCaTs were maintained in high glucose Dulbecco’s modified Eagle’s medium (DMEM) supplemented with 10% fetal bovine serum (FBS), 2 mM glutamine, 100 U/mL penicillin, and 100 μg/mL streptomycin at 37 °C, 5% CO_2_, and used between passages 35 and 48. All experiments were conducted with the aforementioned medium, with FBS content reduced to 0.5%.

### Migration fence assay

A fence migration assay was used to mimic collective cell migration into a wound area^15^. Ibidi® cell culture inserts (Ibidi, Germany) were used as fences by placing devices in the center of each gel and seeding HaCaTs as a confluent monolayer around the insert (7.5 × 10^5^ cells/well for 30 and 100 kPa gels and 1.5 × 10^6^ cells/well for 1 kPa gels due to differences in seeding efficiency on the substrates). After 24 h, the fence devices were lifted and cells were stained with 1 μM calcein AM (Life Technologies) for 15 min at 37 °C. Cell culture plates were imaged by a Molecular Imager^®^ Gel Doc™ XR System (Bio-Rad, Hercules, CA). After imaging, the calcein solution was removed and replaced with 1 mL of media containing 0, 0.1, 1, or 10 ng/mL EGF (Peprotech, Rocky Hill, NJ). Cell imaging and EGF treatment were repeated every 24 h for a total of 4 days. Percent closure was quantified using ImageJ software (NIH, USA).

### Cell tracking

To track single cells within a collective sheet, cells were stained prior to seeding with 5 μM CellTracker™ Green CMFDA (Life Technologies) for 30 minutes, trypsinized, and then mixed at a ratio of 15% stained cells to 85% unstained cells. Cells were plated as described above and allowed to adhere for 24 h. Fences were lifted, cells were treated with 0-10 ng/mL EGF, and cells were tracked for 24 h. To track cells from 24-48 h, the EGF treatment was repeated at 24 h. To track isolated cells, cells were stained with 5 μM CellTracker^TM^ Green, trypsinized, strained through a 0.2 μM filter, and plated at 25,000 cells/cm^2^. Cells were allowed to adhere for 24 h and then treated with EGF for 24 h. To track cells from 24-48 h, the EGF treatment was repeated at 24 h. Time-lapse imaging was performed using an inverted motorized Olympus IX81 microscope equipped with a Hamamatsu CCD ORCA/AG camera, with cells maintained at 37°C, 5% CO_2_ in a humid environment. Brightfield and fluorescent images were captured every 15 min for 24 h for 15 positions per condition at the wound edge for collective migration or throughout the well for isolated cells. Cell tracking was performed using MetaMorph Advanced software (Molecular Devices, Sunnyvale, CA). Cell speed was calculated from individual cell tracks by taking the distance traveled divided by the time interval. Cell persistence was calculated as described previously by taking the shortest distance between start and end points divided by the actual distance the cell traversed^25^.

### Cell proliferation assay

Keratinocytes were seeded and treated as described above for a period of 2 or 4 days. Six hours prior to fixation, 10 μM EdU was spiked into the media. Proliferation was analyzed with the Click-iT® Alexa Fluor® 555 EdU imaging kit (Life Technologies) according to the manufacturer’s protocol. Three images of randomly selected fields of the cell bulk (100+ cells from the edge) and wound edge (within 10–15 cells of edge) were taken for each gel at 20x magnification and averaged for 3 independent gels. The proliferation index was calculated as a percentage by taking the number of proliferating cells divided by the total cell count in each field of view. Cell counting was conducted using ImageJ software.

### Cell area analysis

Keratinocytes were seeded and treated as described above for a period of 2 or 4 days. At the termination of the experiment, cells were rinsed with PBS and fixed with freshly prepared 4% (w/v) paraformaldehyde for 20 min. Cells were permeabilized with 0.1% Triton X-100 for 20 min, blocked with 3% bovine serum albumin for 1 h, and stained with 1 U Alexa Fluor^®^ 546 conjugated-phalloidin (Life Technologies) for 30 min. Images were taken using an Olympus IX51 coupled with Hamamatsu digital camera at 20x magnification. Cell area of individual cells in the bulk (100+ cells from the wound edge) and at the wound edge (<3 cells from the edge) were quantified by ImageJ software.

### Partial least squares regression (PLSR) model

The relationship between single cell behaviors and collective cell migration was analyzed by PLSR. A detailed explanation of the PLSR algorithm, including examples of how to analyze the model results and example problems, is available in a recently published teaching resource^12^. In PLSR, the **X** matrix of independent observations (*i.e.,* individual cell behaviors) is linearly regressed against the **Y** matrix of dependent observations (*i.e.,* collective migration). Similar to a standard linear regression, the PLSR algorithm will find a function of the data in **X** that quantitatively predicts the collective migration in **Y**. As there are more **X** variables than **Y** variables, a standard linear regression cannot be performed; instead, PLSR collapses variables together to form new dimensions called principal components that are weighted linear combinations of the original variables. To develop these components, the co-variation of **X** and **Y** data is used; in essence, the algorithm identifies variables in **X** and **Y** that show a similar pattern of variation and prioritizes that relationship over those that do not vary together. This priority is reflected in the loadings term for each **X** and **Y** variable – similar loadings values reflect similar variation and suggest potential relationships.

Here, an independent block matrix (**X**, dimensions 8 × 8) was generated with each column corresponding to the data for the individual cell behaviors (cell area in the bulk/edge at day 2, proliferation in the bulk/edge at day 2, speed/persistence for 0–24 hours, and speed/persistence for 24–48 hours). Each row represented a different treatment combination, with a total of eight rows corresponding to the four EGF doses at 30 kPa or 100 kPa (1 kPa data was not included in this analysis as there was little collective migration in these conditions). A dependent matrix (**Y**, dimensions 8 × 2) was generated, with the rows corresponding to the same cellular conditions listed above and two columns for wound closure percentage at day 3 and day 4. All data were mean-centered and unit variance scaled and analyzed in SimcaP+ v.12.0.1 (Umetrics, San Jose, CA). A nonlinear iterative partial least squares (NIPALS) algorithm was used and the model was tested for goodness of prediction (Q^2^Y) using a leave-one-out cross validation approach^19^. To examine the impact of proliferation on wound closure, the level of bulk/edge proliferation was set to 0 for conditions in the **X** matrix and the PLSR model was used to predict the effect on wound closure in **Y**. Model predictions were then compared to the experimentally-determined levels of wound closure for aphidicolin-treated cells.

### Proliferation inhibition during fence migration

Keratinocytes were seeded as described above and treated with 4 μg/mL aphidicolin (Cayman Chemicals, Ann Arbor, MI) or vehicle (DMSO, 0.04%). Aphidicolin and EGF treatments were repeated every 24 h for 4 days and percent closure was calculated for each day as described previously. Proliferation inhibition of cells on 30 and 100 kPa substrates treated with 10 ng/mL EGF was confirmed at time points of 1, 2, 3, and 4 days using the proliferation protocol described above. To account for day-to-day variability when comparing to model predictions, the difference in the 30 kPa, 0 ng/mL EGF, vehicle-treated condition relative to the model training set was subtracted from all of the aphidicolin-treated conditions.

### Statistics

All data are presented as mean ± standard deviation (SD). All experiments were performed at least twice to confirm reproducibility. Two-way ANOVA followed by Bonferroni post-test was performed using GraphPad Prism software (La Jolla, California). A p*-*value < 0.05 was considered statistically significant.

## Additional Information

**How to cite this article**: Wickert, L. E. *et al.* Hierarchy of cellular decisions in collective behavior: Implications for wound healing. *Sci. Rep.* **6**, 20139; doi: 10.1038/srep20139 (2016).

## Supplementary Material

Supplementary Information
