# Supplementary material for: Hierarchy of cellular decisions in collective behavior: Implications for wound healing
Source: Sci Rep. 2016 Feb 2;6:20139. doi: 10.1038/srep20139 (PMC4735862; doi:10.1038/srep20139)
Supplement: Supplementary Information [file srep20139-s1.pdf]

Supplementary Information for

Hierarchy of cellular decisions in collective behavior:
Implications for wound healing

Lisa E. Wickert, Shaun Pomerenke, Isaiah Mitchell,
Kristyn S. Masters, Pamela K. Kreeger

Supplemental Methods

Collagen quantification on PAA substrates

Immunostaining was used to verify that collagen concentration was uniform across the different substrate stiffnesses. Gels were first washed with PBS and incubated for 10 min at 25°C with 0.3% H_2O_2 in MeOH. Gels were washed twice with PBS, blocked overnight at 4°C with 3% goat serum in PBS and incubated in 1:1000 anti-collagen, type I (Clone COL-1, Sigma) in 1% goat serum in PBS for 2 h at 25°C. After two washes with PBS, gels were incubated with 1:1000 goat anti-mouse IgG (H+L) HRP conjugated secondary antibody (Thermo Scientific Pierce, Waltham, MA) in 1 % goat serum in PBS, washed twice in PBS, and developed with 1-Step™ Turbo TMB-ELISA (ThermoFisher Scientific) for 5 minutes protected from light. The absorbance was read at 562 nm using a microplate reader (Infinite® M1000 Pro; Tecan, Switzerland).

EGFR immunoblot

Cells were plated as stated above on 1, 30, and 100 kPa substrates and allowed to adhere for 24 h. After lifting the fences, cells were treated with 0 or 10 ng/mL EGF for 15 min or 0-10 ng/mL for EGF dose response on 100 kPa substrates. Cells were then washed once with PBS and lysed with 63.3% glycerol, 2% SDS, 50 mM Tris-HCl (pH 6.8), 10 µg/mL aprotinin, 10 µg/mL leupeptin, 1 µg/mL pepstatin, 1 mM PMSF, 50 U/mL Benzonase nuclease, 1X Phosphatase Inhibitor III, and 5X Phosphatase Inhibitor II (Boston BioProducts, Ashland, MA). Lysates were vortexed, placed on ice for 30 min, and centrifuged at 21,000 x *g* for 15 min at 4°C. Equal volume of lysates were separated via electrophoresis using NuPage® Novex® 4-12% Bis-Tris

Protein Gels (Life Technologies) and transferred onto nitrocellulose membranes (Bio-Rad). Immunoblotting was performed using the Odyssey Infared System (LI-COR, Lincoln, Nebraska) according to manufacturer's suggestions. Briefly, membranes were blocked in Odyssey blocking buffer and membranes were incubated with 1:500 phospho-EGFR (2236, Cell Signaling, Danvers, MA), 1:1000 total EGFR (2232, Cell Signaling), and 1:10,000 GAPDH (14C10, Cell Signaling) overnight in 1% BSA-PBS at 4°C. For detection, secondary antibodies were diluted in 1% BSA-PBS as follows: phospho-EGFR blot with 1:2000 goat anti-mouse IRdye® 800CW, total EGFR with 1:2000 goat anti-rabbit IRdye® 680, and GAPDH with 1:15,000 goat anti-rabbit IRdye® 800CW (LI-COR).

Table S1. Statistical analysis of cell speed and persistence values from Figure 2.

	0 – 24 h		24 – 48 h	
**30 kPa**	Speed	Persistence	Speed	Persistence
**0 vs 0.1** ng/mL EGF	*	NS	NS	*
**0 vs 1** ng/mL EGF	*	*	*	*
**0 vs 10** ng/mL EGF	*	*	*	*
**0.1 vs 1** ng/mL EGF	NS	NS	*	*
**0.1 vs 10** ng/mL EGF	*	*	*	NS
**1 vs 10** ng/mL EGF	*	*	*	NS
**100 kPa**				
**0 vs 0.1** ng/mL EGF	*	NS	*	NS
**0 vs 1** ng/mL EGF	*	*	*	*
**0 vs 10** ng/mL EGF	NS	*	*	*
**0.1 vs 1** ng/mL EGF	*	*	*	*
**0.1 vs 10** ng/mL EGF	*	*	*	*
**1 vs 10** ng/mL EGF	*	*	*	*
**30 vs 100 kPa**				
0 ng/mL EGF	*	NS	NS	NS
0.1 ng/mL EGF	NS	NS	NS	NS
1 ng/mL EGF	*	*	*	NS
10 ng/mL EGF	*	*	*	*

* indicates $p < 0.05$ and NS indicates no significant difference by two-way ANOVA followed by a Bonferroni post-test.

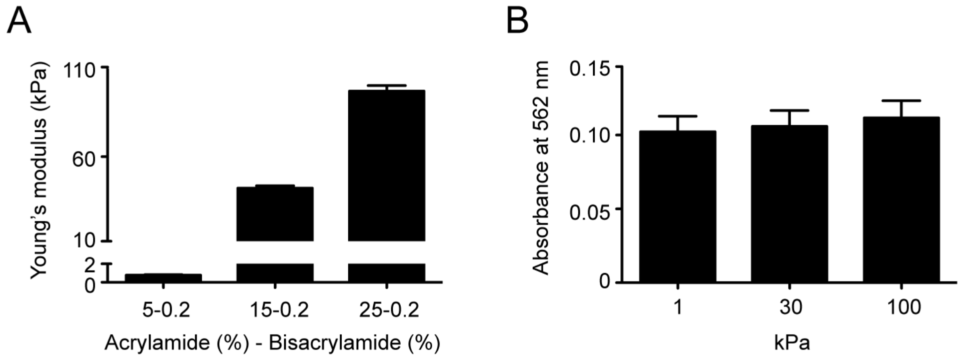

Figure S1. Mechanical properties of PAA gels and quantification of collagen modification. **(A)** Young's moduli of PAA gels with indicated acrylamide (%)-bisacrylamide (%) ratio. **(B)** Quantification of collagen tethered to PAA gels. Data represented as average $\pm$ SD, n=3.

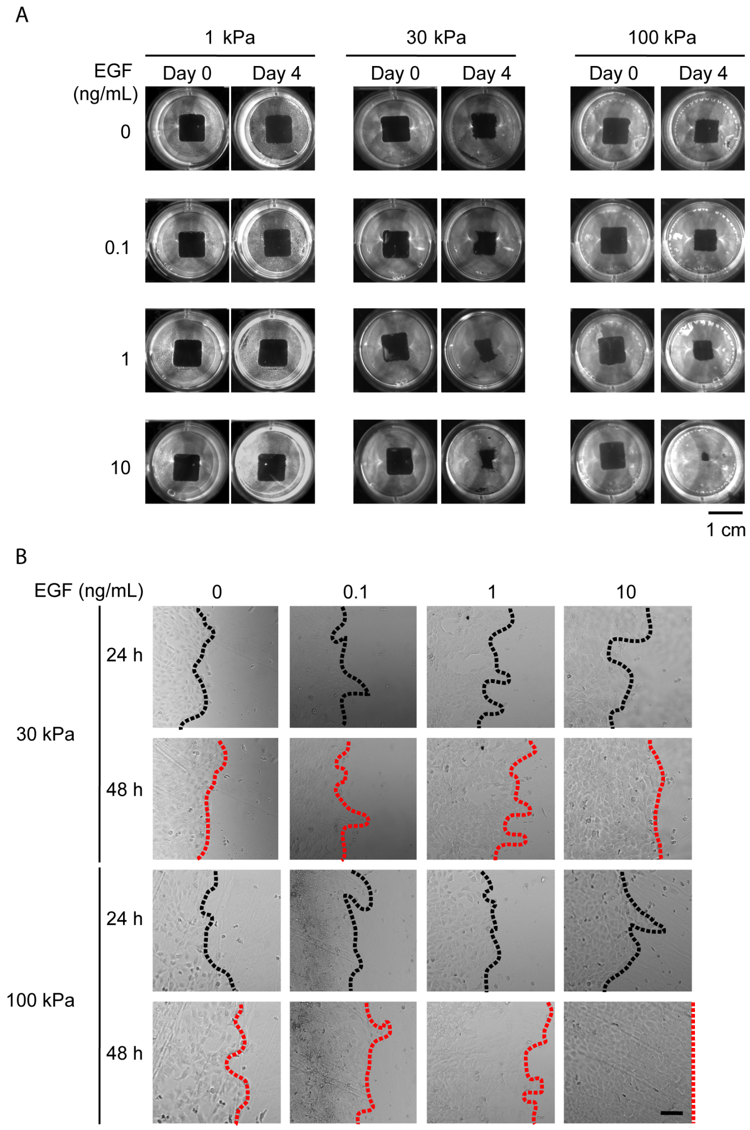

Figure S2. Collective migration imaging. (A) Representative calcein images of collective HaCaTs on 1, 30, and 100 kPa PAA gels at day 0 and day 4 treated with increasing doses of EGF. **(B)** Representative brightfield images at 10X magnification from collective migration cell tracking at 24 and 48 h after fence removal on 30 and 100 kPa substrates treated with increasing doses of EGF. Black dashed line indicates wound edge starting position and red dashed line indicates wound edge ending position for the 24-48 h timeframe. Scale bar = 200 μm

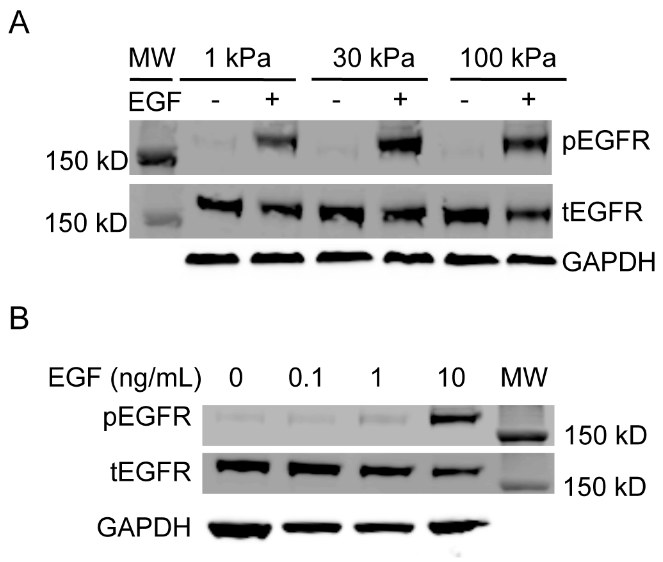

Figure S3. Effect of stiffness and EGF dose on EGFR phosphorylation. Representative Western blots of phosphorylated EGFR, total EGFR, and GAPDH on **(A)** 1, 30, and 100 kPa PAA gels, -/+ 10 ng/mL EGF for 15 min and **(B)** 100 kPa PAA gels, 0-10 ng/mL EGF for 15 min. MW = molecular weight.

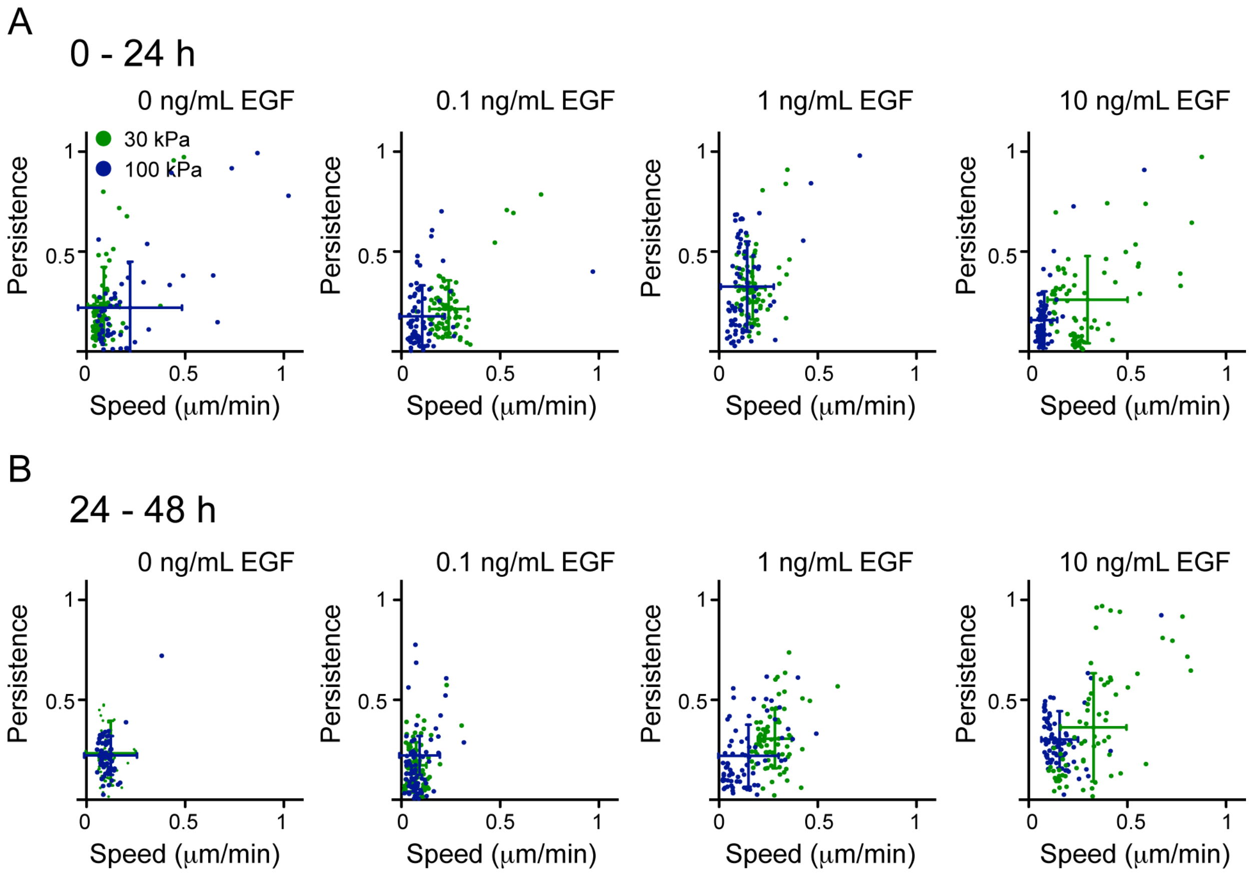

Figure S4. Speed and persistence of individual keratinocytes in isolation. Cell speed vs. persistence for individual cells tracked for (A) 0-24 h and (B) 24-48 h after plating on 30 and 100 kPa PAA gels treated with increasing doses of EGF. Data are presented as a point for each individual cell, error bars represent population average (intersection) and SD, n = 70 for each condition.

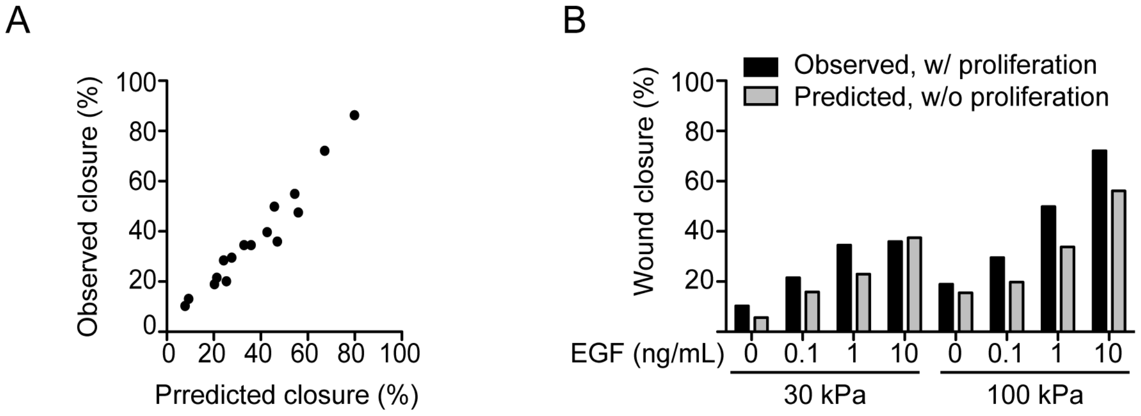

Figure S5. PLSR model for collective migration of keratinocytes. (A) Single component model for day 3 and 4 of wound closure. **(B)** Wound closure (%) at day 3 observed in model training set with proliferation (black) vs. model predicted effect of complete inhibition of proliferation (gray).

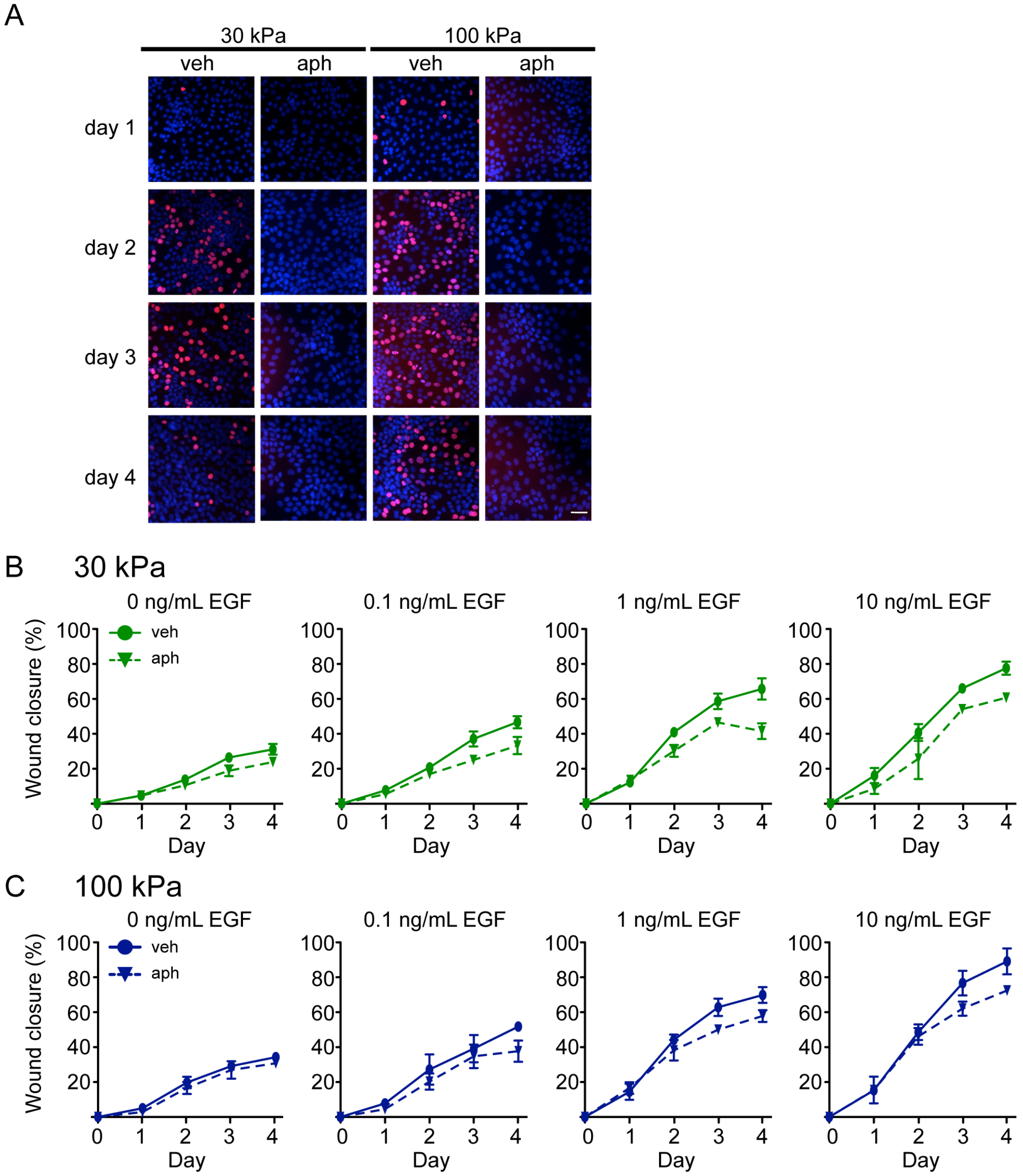

Figure S6. Proliferation inhibition during collective migration. (A) Representative staining of EdU positive cells (red) and total cells (DAPI-blue) with 10 ng/mL EGF on 30 and 100 kPa PAA gel treated with DMSO (veh) or 4 μg/mL aphidicolin (aph) at days 1, 2, 3, and 4 of collective migration. Wound closure area relative to day 0 for a fence migration assay performed on **(B)** 30 and **(C)** 100 kPa PAA gels treated with vehicle or aphidicolin and stimulated with increasing doses of EGF. Data presented as average ± SD, n = 3. Scale bar = 50 μm.
